# Predictive Value of Globulin to Prealbumin Ratio for 3-Month Functional Outcomes in Acute Ischemic Stroke Patients

**DOI:** 10.1155/2022/1120192

**Published:** 2022-03-17

**Authors:** Chunjian Li, Chenguang Yang, Junyan Zhu, Honghao Huang, Jiahui Zheng, Xueting Hu, Wenjing Pan, Fangyue Sun, Tian Zeng, Haojie Qiu, Zerui Jiang, Yiqun Chen, Yilin Chen, Guangyong Chen, Yiyun Weng

**Affiliations:** ^1^Clinical Laboratory, The Third Affiliated Hospital of Wenzhou Medical University, Wenzhou, China; ^2^School of the First Clinical Medical Sciences, Wenzhou Medical University, Wenzhou, China; ^3^Department of Medical Ultrasonics, The First Affiliated Hospital of Zhejiang Chinese Medical University, Hangzhou, China; ^4^Department of Otolaryngology, The First Affiliated Hospital of Wenzhou Medical University, Wenzhou, China; ^5^School of the Second Clinical Medical Sciences, Wenzhou Medical University, Wenzhou, China; ^6^Department of Neurology, The Third Affiliated Hospital of Wenzhou Medical University, Wenzhou, China; ^7^Department of Neurology, The First Affiliated Hospital of Wenzhou Medical University, Wenzhou, China

## Abstract

**Objective:**

We aimed to evaluate and compare the association between globulin to albumin ratio (GAR) and globulin to prealbumin ratio (GPR) and 3-month functional prognosis of acute ischemic stroke (AIS) patients receiving intravenous thrombolysis therapy.

**Methods:**

234 AIS patients undergoing intravenous thrombolysis were retrospectively enrolled with acute ischemic stroke from February 2016 to October 2019. Blood sample was collected within 24 h after admission. Poor outcome was defined as the modified Rankin Scale (mRS) ≥ 3 and a favorable outcome as mRS < 3. Severe stroke was defined as the National Institutes of Health Stroke Scale (NIHSS) score > 10 on admission. Student's *t*-test, Mann–Whitney *U* test, Chi-square test, logistics' regression analysis, and receiver operating characteristic (ROC) analysis were performed.

**Results:**

Patients with poor functional outcome had higher GAR and GPR levels compared with favorable functional group (*p* = 0.001, *p* < 0.001, respectively). Severe stroke was also associated with these two increasing variables. After adjustment for confounding factors, multivariate logistic regression analysis indicated that GPR was an independent indicator predictor of AIS.

**Conclusions:**

The 24 h GPR level can predict the 3-month functional outcome in AIS patients accepting recombinant tissue plasminogen activator (r-tPA) intravenous thrombosis.

## 1. Introduction

Stroke has become the most major cause of death and adult disability in China and the second leading cause of death in the world [1–3]. Recombinant tissue plasminogen activator (r-tPA) can recanalize occluded blood vessels as soon as possible, and intravenous r-tPA therapy is recommended as the first-line treatment for acute ischemic stroke (AIS) patients within 4.5 h from symptom onset. Nonetheless, there is still a large proportion of patients who have received thrombolytic therapy without symptoms improved. Early judgment of the prognosis of AIS can help doctors to stratify the outcomes of patients and formulate appropriate treatment and rehabilitation programs. The prognosis of patients with AIS is related to many factors. Among them, inflammation and nutritional status in the acute phase have an important impact on the prognosis [4, 5].

Globulin to albumin ratio (GAR) is a common indicator of inflammation and nutritional status [6]. Serum albumin level is regarded as a predictor of ischemic stroke outcome [7]. Globulin is a major protein produced by immune organs, which reflects the inflammatory and immune status of the human body [8]. However, there are few studies on the relationship between GAR and stroke prognosis. Compared to albumin, prealbumin has a lower plasma concentration and a shorter half-life, which can more accurately reflect the nutritional status of patients in acute phase [9]. This indicates that prealbumin is a more acute nutritional marker than albumin. A study has shown that globulin to prealbumin ratio (GPR) can be used to predict the prognosis of gastric cancer, but there is no study on the relationship between the ratio of globulin to prealbumin and the prognosis of acute ischemic stroke [10]. The purpose of this study was to evaluate and compare the relationship between GAR or GPR and 3-month functional prognosis of AIS patients receiving intravenous thrombosis therapy.

## 2. Methods

### 2.1. Study Population

**Figure 1:**
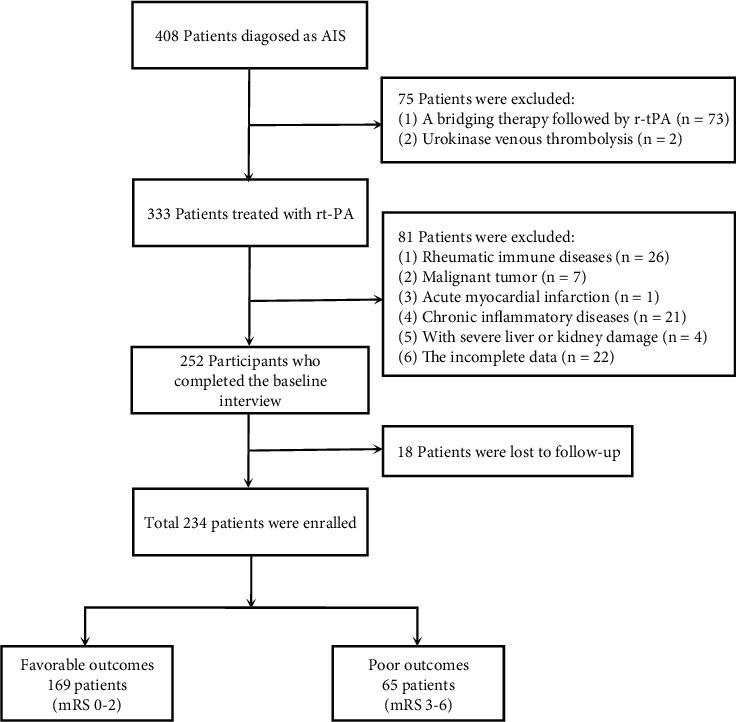
Flow chart for patients' selection.

This retrospectively study was conducted among 408 patients with AIS from February 2016 to October 2019. All patients had accepted treatment at the Third Affiliated Hospital of Wenzhou Medical University.  Figure 1 shows how patients were selected. The exclusion criteria were as follows: (1) patients treated with bridging therapy followed by r-tPA; (2) with urokinase venous thrombolysis; (3) with rheumatic immune diseases; (4) with malignant tumor; (5) with acute myocardial infraction; (6) with severe liver or kidney damage; (7) with chronic inflammatory disease; and (8) with incomplete data and missing follow-up data. After the exclusion, 234 patients were finally enrolled into the study and 18 patients died during the three-month follow-up. The study was approved by the Ethics Committee of the Third Affiliated Hospital of Wenzhou Medical University and was conducted in accordance with the Declaration of Helsinki. All subjects had signed a written informed consent form.

### 2.2. Data Collection

The demographic and baseline data of AIS patients about age, sex, hypertension, diabetes, history of stroke, hyperlipidemia, and atrial fibrillation were obtained from medical records. Blood chemistry analyses were performed within 24 h on admission. GAR was calculated as globulin/albumin and GPR as globulin/prealbumin.

National Institutes of Health Stroke Scale (NIHSS) scores at admission were used to evaluate the severity of ischemic stroke by stroke neurologists. Severe stroke was defined as NIHSS > 10 [11]. Two experienced neurologists followed up patients or their family by telephone interviews. 3-month functional outcomes of AIS patients were assessed using the modified Rankin Scale (mRS). Poor functional outcome was defined as mRS ≥3 [12].

### 2.3. Statistical Analysis

Statistical analyses were performed by using SPSS 25.0 software (SPSS Inc., Chicago, IL, USA). The continuous variables of normal distribution were expressed as mean ± standard deviation (SD), while variables of nonnormal distribution were expressed as median (interquartile range [IQR]). Frequency and percentage expressed the classified variables. All subjects were divided into two groups according to the 3-month mRS score. Independent sample *t*-test was used to compare the continuous variables of normal distribution. Mann–Whitney *U* test was used to compare nonnormally distributed variables. Pearson chi-square test or Fisher exact test was used to compare categorical variables. The cut-off values for GAR and GPR were determined by receiver operating characteristic (ROC) curve. The optimal cut-off values were evaluated using Youden index (Youden index = maximum [sensitivity − (1 − specifity)]). To show the predictive ability of indicators, the area under the curve (AUC) of poor outcomes was also calculated. The variables with *p* < 0.05 in univariate analysis were included in further multivariate logistic regression. A *p* value <0.05 was considered to be statistically significant.

## 3. Results

### 3.1. Baseline Characteristics of the Study Subjects

**Figure 2:**
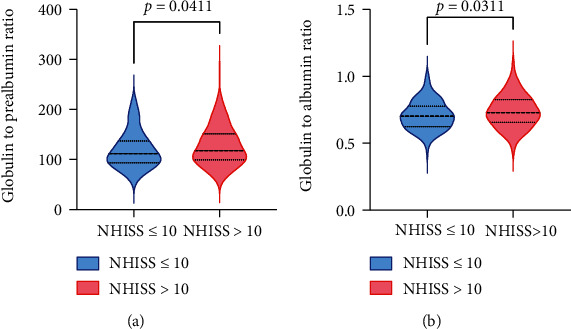
Correlation between GPR (a) and GAR (b) levels and disease severity according to NIHSS scores in AIS patients. Bar represents median with interquartile range.

Baseline characteristic and 24 h laboratory indexes were described in Table 1. After exclusion, a total of 234 patients were included in the analysis. Among those patients, patients with poor outcome (*n* = 65) were on average older and had more often a risk profile about hypertension, atrial fibrillation, and history of stroke. Meanwhile, they had higher baseline NIHSS score, which revealed that their neurological functions were worse damaged. By comparing the 24 h laboratory indexes, prealbumin levels increased in the favorable outcome group, but they had lower levels of WBC, GPR, and GAR. As Figure 2 shows, severe stroke patients all had higher levels of GPR and GAR.

### 3.2. Association Between GPR and GAR and Functional Outcomes

**Figure 3:**
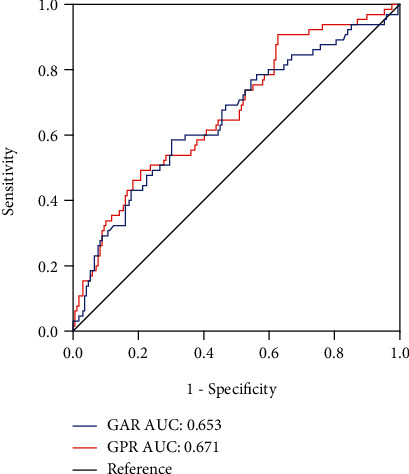
Receiver operating characteristic curve analysis of the globulin to prealbumin ratio (GPR) and the globulin to albumin ratio (GAR) in poor outcome patients with AIS.

**Figure 4:**
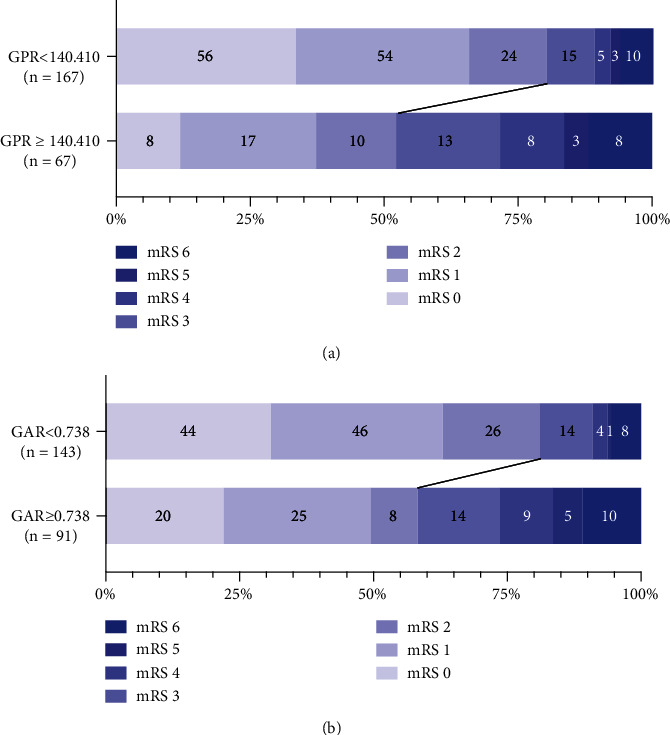
Distribution of 3-month mRS scores in optimal cut-off value of the globulin to prealbumin ratio (GPR) (a) and the globulin to albumin ratio (GAR) (b). Numbers in bars are absolute numbers.

To further investigate the relationship between GPR or GAR and 3-month outcomes, ROC curves were calculated (Figure 3). The optimal cut-off values of GAR and GPR as indicators for diagnosis of poor outcome were 0.738 and 140.41, which yielded a sensitivity of 58.5% and a specificity of 69.8% (AUC = 0.653; 95% CI, 0.589-0.714; *p* < 0.001) and a sensitivity of 58.5% and a specificity of 69.8% (AUC = 0.671; 95% CI, 0.607-0.731; *p* < 0.001), respectively. By using the cut-off value to categorize patients, Figure 4 visualizes the distribution of functional outcomes at 3 months. According to the GPR cut-off value, the frequency of mRS score ≤ 3 in the low GPR group was lower than that of the high GPR group, and the frequency of mRS ≥ 3 was higher than that of the high GPR group. A similar frequency distribution could be seen in the GAR classification.

### 3.3. GPR as an Independence Predictor of AIS

Univariate logistic regression was performed to investigate the correlation between GAR and GPR and 3-month poor outcome (Table 2). We found that the evaluated GPR levels were significantly associated with 3-month poor outcomes. GAR also showed analogous correlation. The indicators with significant *p* value (*p* < 0.05) in univariate analysis comprising age, hypertension, history of stroke, atrial fibrillation, WBC, BUN (blood urea nitrogen), and baseline NIHSS score were incorporated into multivariate logistic regression models. For further study, multivariate logistic regression analysis was performed (Table 3). Model 1 was simply adjusted for age, and we found that the aOR value of GPR slightly decreased compared to univariate logistic analysis. Adjusted for age, hypertension, history of stroke, atrial fibrillation, BUN, and WBC, the *p* value of GPR was still outstanding (aOR 1.013, 95% CI 1.003–1.023, *p* = 0.010) in the model 2. After adjustment for all significant indicators mentioned above in the model 3, GPR was independently associated with poor outcomes (aOR 1.013, 95% CI 1.002–1.024, *p* = 0.017). The significant correlation between GAR and short-term poor outcome disappeared in the adjusted models.

## 4. Discussion

As far as we know, our study is the first one to investigate the correlation between GPR and the outcomes of AIS patients receiving r-tPA intravenous thrombolysis therapy. The main findings of this retrospective study are as follows: (1) we found that the evaluated 24 h GPR and GAR were associated with 3-month poor outcomes. (2) Patients in the high GPR group tended to have higher mRS scores. (3) After being adjusted for confounding factors, GPR was an independence predictor of AIS prognosis, but the significance of GAR was no longer outstanding.

Inflammatory response and immune mediation are involved in the process of lipid deposition in atherosclerotic arteries [13]. Atherosclerosis is an independent risk factor for ischemic stroke. In the occurrence of ischemic stroke, monocyte macrophages and T lymphocytes are stimulated to produce inflammatory cytokines, such as tumor necrosis factor *α* (TNF-*α*), interleukin 6 (IL-6), and interleukin 1 (IL-1), which have immunomodulatory function [14]. These proinflammatory cytokines can promote the serum globulin concentrations, but the exact mechanism of the effect on prognosis is not clear. The possible mechanism is that IL-6, IL-1, TNF-*α*, and other cytokines can be applied to the liver and induce the liver to synthesize positive acute phase reactants through acute phase protein gene, many of which are globulins, such as C-reactive protein, *α*2-macroglobulin, prothrombin, fibrinogen, and serum amyloid A [15]. Previous study found an association between high C-reactive protein levels within 24 hours of admission and 3-year mortality. [16] Meanwhile, IL-6, IL-1, and TNF-*α* can strongly upregulate matrix metalloproteinase 9 (MMP-9) [17–20]. Study found that MMP-9 take part in tPA-associated hemorrhage, so the uncontrolled activation of MMP after tPA reperfusion can degrade key proteins in the cerebrovascular system, thereby damaging the structural integrity of the vessels and ultimately leading to vascular rupture [21]. Another possible mechanism is poststroke immunodepression. In the acute phase, the immune system will be overactivated within 1-2 days, but after that, the poststroke immunodepression will increase the possibility of infection which can lead the poor functional outcome [22].

Evidence showed that malnutrition was an independence risk factor for mobility and mortality in AIS patients [5, 23, 24]. Compared with nourished patients, malnourished patients had a higher frequency of gastrointestinal bleeding, bedsores, pneumonia, and other infections [5, 24]. Serum prealbumin, also known as transthyretin, is a tetramer composed of four identical subunits. Its main function is to transport thyroxine and vitamin A, and it can promote lymphocyte maturation and enhance immunity. The prealbumin has a low concentration with a half-life of 2 days, which can accurately and sensitively reflect the internal protein synthesis in the body and is one of the important indicators for the evaluation of nutritional status in the world [25]. Because its half-life is shorter than that of albumin (18 to 23 days), it better reflects the balance of nutrient intake and slight changes in liver synthesis and catabolism. Some studies have also suggested that serum prealbumin has a protective effect on neurons, and the main mechanism may be the reverse transport of neurotransmitters promoted by prealbumin, and the growth of axons and regeneration and repair of neurons promoted by macroprotein-dependent internalization [26–28]. The possible mechanism is as follows: (1) the prealbumin can reflect the body's immune function to a certain extent. When immune function decreases, the current albumin levels drop, increasing the risk of poor prognosis. (2) Decreased prealbumin in acute phase indicates protein malnutrition and the inhibition of the synthesis of acute phase reactive protein will lead to the weakening of the neutralization effect of toxic metabolites generated by ischemic infarction focus cells. (3) Prealbumin may be involved in the repair and regeneration of injured and necrotic neurons, so decreased prealbumin level is not conducive to the growth and functional recovery of axons of injured neurons.

The high mortality rate and disability rate of stroke cause great social burden and clinical burden. [29] Predicting the prognosis of stroke with biochemical indicators has become a research hotspot in this field. Zhou et al.'s study found the predictive value of decreased A/G levels in poor outcome of acute ischemic stroke. [30] In our study, we used prealbumin and globulin to evaluate the nutritional status and inflammatory level of patients and found that they can well predict the 3-month prognosis of patients. At the same time, it also revealed the relationship between inflammation and malnutrition and functional prognosis. This study suggested that both prealbumin and hyperglobulin can lead to poor prognosis in patients with acute ischemic stroke. Monitoring and intervention of prealbumin and hyperglobulin in patients with acute ischemic stroke may improve their prognosis. Recently, study showed that inhibition of inflammatory response can improve the neurological deficit in patients with ischemic stroke [22]. High dose albumin therapy is also considered to improve patients' behavioral function within the effective treatment time window, suggesting that malnutrition may be a potential target [31]. However, Martin et al.'s study opposes the therapeutic effect of albumin [32]. The relationship between prealbumin and functional outcome found in our study may provide new ideas for clinical treatment.

However, there are some limitations in our study. First, patients in a single study center were enrolled in this study, which might cause bias in the results. Secondly, it is a retrospective study with small sample size. The small sample size maybe the reason why GAP did not show significance in the multivariate analysis. More detailed multicenter prospective study needs to be conducted to further clarify the issue. What is more, we lack the data of fraction volume, but there is a high correlation between baseline NIHSS score and infarct volume. To some extent, NIHSS score can be used as a substitute for infarct volume [33]. Fourthly, prealbumin was only tested within 24 h on admission, so dynamic analysis cannot be performed to reflect the change of GPR level during hospitalization. Finally, the study only included patients receiving thrombolytic therapy and did not study the influence of other treatment methods on indicators.

## 5. Conclusion

The 24 h GPR level can predict the 3-month poor functional outcome in AIS patients accepted r-tPA intravenous thrombosis.

## Figures and Tables

**Table 1:** Comparison of clinical characteristics between favorable and poor outcome.

Variables	Favorable outcome	Poor outcome	*p* value
	**(** *n* = 169)	**(** *n* = 65)	
Demographic characteristics			
Age (years)	66 (55.5-75)	75 (67-82.5)	<0.001
Sex (male, *n* %)	112 (66.3)	38 (58.5)	0.265
Vascular risk factors, (*n* %)			
Hypertension (*n* %)	107 (63.3)	51 (78.5)	0.027
Diabetes (*n* %)	73 (43.2)	30 (46.2)	0.683
Hyperlipidemia (*n* %)	52 (30.8)	22 (33.8)	0.650
History of stroke (*n* %)	15 (8.9)	14 (21.5)	0.008
Atrial fibrillation (*n* %)	42 (24.9)	34 (52.3)	<0.001
Clinical data			
Baseline NIHSS	6.00 (4.00-9.00)	13.00 (8.00-18.00)	<0.001
Laboratory indexes			
WBC (×109/L)	7.08 ± 1.85	8.66 ± 2.58	<0.001
RBC (×1012/L)	4.41 ± 0.49	4.28 ± 0.53	0.078
Platelet (×109/L)	197.35 ± 54.89	197.88 ± 57.35	0.948
Albumin (g/L)	38.18 ± 2.92	38.06 ± 3.21	0.788
Globulin (g/L)	26.70 ± 3.90	28.95 ± 4.94	<0.001
Prealbumin (g/L)	0.23 (0.20-0.27)	0.22 (0.18-0.25)	0.005
UA (*μ*mol/L)	331.48 ± 81.44	317.78 ± 98.03	0.278
BUN (mmol/L)	4.60 (3.95-5.67)	5.28 (4.03-6.22)	0.060
TC (mmol/L)	4.53 (4.01-5.24)	4.67 (3.69-5.61)	0.601
HDL-C (mmol/L)	1.07 (0.94-1.28)	1.03 (0.89-1.34)	0.660
LDL-C (mmol/L)	2.90 ± 0.90	3.07 ± 1.07	0.219
Apo-A1 (g/L)	1.29 (1.17-1.43)	1.23 (1.10-1.49)	0.357
Apo-B1 (g/L)	0.92 (0.78-1.06)	0.93 (0.81-1.12)	0.453
GPR	111.92 (93.74-135.75)	136.32 (106.05-174.08)	<0.001
GAR	0.70 ± 0.11	0.77 ± 0.13	0.001

Abbreviations: NIHSS: National Institute of Health Stroke Scale; WBC: white blood cell count; UA: uric acid; RBC: red blood cells; BUN: blood urea nitrogen; TC: triglyceride; HDL-C: high density lipoprotein-cholesterol; LDL-C: low density lipoprotein-cholesterol; Apo-A1: apolipoprotein-A1; Apo-B1: apolipoprotein-B1; GPR: globulin to prealbumin ratio; GAR: globulin to albumin ratio.

**Table 2:** Univariate logistic regression analyses for prognosis.

Variable	Univariate logistic regression		
	OR	95% CI	*p* value
Age	1.069	1.039-1.100	< 0.001
Sex	1.396	0.776-2.512	0.266
Hypertension	2.111	1.081-4.121	0.029
Diabetes	1.127	0.634-2.003	0.683
Hyperlipidemia	1.151	0.626-2.116	0.650
History of stroke	2.818	1.274-6.236	0.011
Atrial fibrillation	3.316	1.822-6.036	< 0.001
WBC	1.402	1.210-1.625	< 0.001
Albumin	0.987	0.897-1.086	0.787
RBC	0.597	0.335-1.063	0.080
Platelet	1.000	0.995-1.005	0.948
Globulin	1.129	1.054-1.210	0.001
Prealbumin^a^	0.913	0.859-0.969	0.003
UA	0.998	0.995-1.002	0.278
BUN	1.231	1.008-1.505	0.042
TC	1.103	0.851-1.429	0.461
HDL-C	1.089	0.370-3.199	0.877
LDL-C	1.206	0.894-1.626	0.220
Apo-A1	0.836	0.241-2.900	0.778
Apo-B1	1.921	0.645-5.724	0.241
NIHSS	1.240	1.162-1.324	< 0.001
GPR	1.017	1.009-1.025	< 0.001
GAR	100.787	7.857-1292.809	0.001

Abbreviations: WBC: white blood cell count; UA: uric acid; RBC: red blood cells; BUN: blood urea nitrogen; TC: triglyceride; HDL-C: high density lipoprotein-cholesterol; LDL-C: low density lipoprotein-cholesterol; Apo-A1: apolipoprotein-A1; Apo-B1: apolipoprotein-B1; NIHSS: National Institute of Health Stroke Scale; GPR: globulin to prealbumin ratio; GAR: globulin to albumin ratio. ^a^OR is intended for per 0.01-point increase of prealbumin.

**Table 3:** Adjusted models for prognosis at 3 months.

Variable	GPR			GAR		
	aOR	95% CI	*p* value	aOR	95% CI	*p* value
Model 1	1.012	1.003-1.021	0.006	14.791	0.951-229.955	0.054
Model 2	1.013	1.003-1.023	0.010	17.795	0.870-363.843	0.062
Model 3	1.013	1.002-1.024	0.017	26.965	0.901-807.307	0.057

Model 1 is adjusted for age. Model 2 is adjusted for age, hypertension, history of stroke, atrial fibrillation, BUN (blood urea nitrogen), and WBC (white blood cell count). Model 3 is adjusted for NIHSS score, age, hypertension, history of stroke, atrial fibrillation, BUN, and WBC.

## Data Availability

The data that support the findings of this study are available from the corresponding author on reasonable request.
